# Higher frequency of specific periodontopathogens in hypertensive patients. A pilot study

**DOI:** 10.1590/0103-6440202204914

**Published:** 2022-10-21

**Authors:** Taciane Menezes da Silveira, Caroline Fernandes e Silva, Rodrigo de Almeida Vaucher, Patrícia Daniela Melchiors Angst, Maísa Casarin, Natália Marcumini Pola

**Affiliations:** 1 Graduate Program in Dentistry, Federal University of Pelotas, Pelotas, RS, Brazil.; 2 Center of Chemical, Pharmaceutical and Food Sciences, Federal University of Pelotas, Pelotas, RS, Brazil.; 3 Division of Periodontics, School of Dentistry, Federal University of Rio Grande Do Sul, Porto Alegre, RS, Brazil.; 4 Division of Periodontics, Graduate Program in Dentistry, Federal University of Pelotas, Pelotas, RS, Brazil.

**Keywords:** periodontitis, hypertension, blood pressure, bacteria, real-time polymerase chain reaction

## Abstract

Periodontitis and arterial hypertension are two of the pathologies with the highest global prevalence; evidence reported so far has been favorable to an association between them. This cross-sectional study aimed to evaluate and compare the microbiological counts of hypertensive and normotensive patients with periodontitis. Sociodemographic, behavioral, systemic health data and periodontal clinical parameters were assessed. Counts of *A. actinomycetemcomitans, P. intermedia, P. gingivalis* and *F. nucleatum* were performed by real-time polymerase chain reaction using subgingival biofilm samples. Thirty-eight patients were included in this preliminary analysis, divided into two groups: Normotensive Group (NG) (n = 14) and Hypertensive Group (HG) (n = 24). Patients diagnosed with periodontitis composed both groups. Data analysis was performed with significance level of 5%. There was no significant difference between groups for clinical periodontitis diagnosis. In addition, hypertensive individuals had higher *P. intermedia, P. gingivalis,* and *F. nucleatum* counts when compared to normotensive individuals. The parameters probing pocket depth, bleeding on probing, and *A. actinomycetemcomitans* count did not presented statistical differences between groups. With these preliminary results, it can be concluded that the presence of arterial hypertension may be associated with a greater quantity of periodontopathogenic bacterial of some species in individuals with periodontitis.

## Resumo

A periodontite e a hipertensão arterial são duas das patologias com maior prevalência global, as evidências relatadas até o momento têm sido favoráveis ​​a uma associação entre elas. Este estudo transversal teve como objetivo avaliar e comparar contagem microbiológicas de pacientes hipertensos e normotensos com periodontite. Dados sociodemográficos, comportamentais, de saúde sistêmica e parâmetros clínicos periodontais foram avaliados. Contagens de *A. actinomycetemcomitans, P. intermedia, P. gingivalis* e *F. nucleatum* foram realizadas por reação em cadeia da polimerase em tempo real utilizando amostras de biofilme subgengival. Trinta e oito pacientes foram incluídos nesta análise preliminar, divididos em dois grupos: Grupo Normotenso (GN) (n = 14) e Grupo Hipertenso (GH) (n = 24). Pacientes diagnosticados com periodontite compuseram os dois grupos. A análise dos dados foi realizada com nível de significância de 5%. Não houve diferença significativa entre os grupos para o diagnóstico clínico de periodontite. Além disso, os hipertensos apresentaram maior contagem de *P. intermedia, P. gingivalis* e *F. nucleatum* quando comparados aos normotensos. Os parâmetros profundidade de sondagem, sangramento à sondagem e contagem de *A. actinomycetemcomitans* não apresentaram diferenças estatísticas entre os grupos. Com esses resultados preliminares, pode-se concluir que a presença de hipertensão arterial pode estar associada a uma maior quantidade de bactérias periodontopatogênicas de algumas espécies em indivíduos com periodontite.

## Introduction

Periodontitis is a complex polymicrobial inflammatory pathology associated with dysbiotic plaque biofilms and identified by the gradual tooth support loss ^1^. The prevalence of severe chronic periodontitis in 2015 has reached 616 million, and it is estimated that the worldwide ranges between 20 and 50% of the population ^2^. Considering its high occurrence, the identification of periodontitis as a potential risk factor of various systemic diseases is extensively investigated. In this scenario, studies have shown that periodontitis could be associated with cardiovascular diseases (CVD) ^3^. Regarding modifiable risk factors for CVDs, arterial hypertension affects 30 to 45% of the adult population. It is also estimated that by 2025 a third of the world’s population could be hypertensive ^4^.

The literature has reported a significant association between periodontitis and hypertension (HT) ^5^. Periodontal disease markers as gingival bleeding, probing pocket depth, and attachment loss were associated with raised arterial blood pressure and HT in the US adult population ^6^. Besides, there are periodontitis pathologic pathways that may contribute to a state of widespread inflammation, acting as a substrate for an increase in blood pressure ^5^. The periodontal bacterial load and transient bacteremia, the subsequent immune response, the widespread systemic inflammation, and glucose - lipid metabolism could be reported as some of these pathways ^7^. On the other hand, blood pressure elevation could affect the membrane of periodontal blood vessels, inducing the rupture of small arteries and promoting gingival bleeding and alveolar support changes ^7^. 

Furthermore, certain periodontopathogenic bacterial species may affect and be influenced by the individual's systemic condition. *A. actinomycetemcomitans* can modify atherosclerosis courses, such as lipoprotein serum concentration, endothelial permeability, and binding of lipoproteins in the arterial intima ^3^. Another bacterial investigation involving patients with heart valve disease showed that *P. intermedia* was found in the cardiac valve samples of 19.1% of the participants, who also had a high rate of periodontitis ^8^. The levels of circulating antibodies and protein antigens to *P. gingivalis* shows correlate to attachment loss, as a marker for periodontitis and cardiovascular disease ^9^. However, no previous study has compared the counts of periodontopathogens between hypertensive and normotensive individuals.

The evidence of the interrelationships between periodontitis and HT is still conflicting ^10^, which justifies the need to investigate the mechanisms and factors that could strengthen or weaken this association. Therefore, this cross-sectional study presents an unprecedented investigation that evaluate and compare the microbiological counts of hypertensive and normotensive patients with periodontitis.

## Material and methods

### Study design and ethical considerations

The study is reported according to the guidelines proposed in the STROBE statements ^11^. Individuals invited to participate in the study were informed about the objectives, risks, and benefits. Informed consent was obtained from all participants that were consecutively included in the study. The research protocol was approved by the Ethics Committee in Research of the Medicine School of Federal University of Pelotas 1, 043, 441.

### Patient recruitment

This study was conducted with individuals from Pelotas/RS, Brazil. Subjects recruited to this study included patients with and without a confirmed diagnosis of hypertension, and with periodontitis. The sample was partially recruited at a public hospital from the Federal University of Pelotas - UFPel (Amilcar Gigante Health Research Center), and by publicity on medical clinics and at social media on the Internet (e.g. Instagram and Facebook), to contemplate the hypertensive group (HG) and normotensive group (NG). The recruitment period comprised of April 2018 to July 2019, and individuals were included in the sample as they were identified.

### Eligibility criteria

Patients aged between 35 and 75 years old, without distinction of sex, presenting at least 8 permanent teeth, and with the diagnosis of mild, moderate, or severe periodontitis were included ^1^. It was adopted specific inclusion criteria for hypertension diagnoses according to arterial pressure gauging ^12^. All patients included should present systolic blood pressure <130 mmHg and diastolic blood pressure <80 mmHg. Normotensive patients should have a non-history of arterial hypertension. The hypertensive patients were those who presented hypertension diagnoses established more than six months previously the study beginning and in current use of anti-hypertensive medicines. The exclusion criteria were smoking habit; obesity; diabetes mellitus; metabolic syndrome; autoimmune diseases; chronic kidney diseases; chronic obstructive pulmonary diseases; pregnancy; use of antibiotics, anti-inflammatory or immunosuppressive medications in the last 6 months; history of periodontal treatment in the last 6 months; current use of a fixed orthodontic appliance. Self-declared hypertensive patients, with decompensated blood pressure (BP) and individuals who did not reported the use of medication to control BP; and/or individuals with prehypertension, history of stroke, or myocardial infarction, were referred for cardiac evaluation and were not included in the study.

### Data collection

The data collection was performed in two sessions by two not blinded examiners. First, a questionnaire to investigate the dental and general health, as well as demographic, behavioral, and socioeconomic data of the individuals were applied by trained examiners. Systemic conditions report and the medications used by normotensive and hypertensive patients were also requested. Patients underwent physical and periodontal examinations. In a second moment, it was performed the subgingival biofilm samples collection, prophylaxis, and oral hygiene guidance. A blood test was performed by a private laboratory (Sancti Laboratory of Clinical Analysis, Pelotas, RS, Brazil) to evaluate plasma levels of the following parameters: fasting glucose, triglycerides, cholesterol HDL, LDL, and total, C-reactive protein (CRP), and fibrinogen.

For physical exam, the weight, height, and waist circumference of all participants were collected according the protocols from the Center for Disease Control and Prevention ^13^. BP was measured using a calibrated digital device (HEM-7113, OMRON, São Paulo, SP, Brazil), in the patient's left arm in the sitting position, after 5 minutes of rest. The measurement was performed three times, with an interval of two minutes between each measurement, and the recorded value corresponded to the average of the three values.

### Periodontal status

The periodontal parameters as visible plaque index (VPI), marginal bleeding index (MBI), plaque retentive factor (PRF - supragingival calculus, poorly adapted restorations and prostheses, and coronary fractures), probing pocket depth (PPD), bleeding on probing (BOP), and clinical attachment loss (CAL) were performed by two calibrate dentists. The intra-class correlation coefficient (ICC) values were 0.95 (PPD) and 0.98 (CAL) for the examiner 1 and 0.94 (PPD) and 0.98 (CAL) for the examiner 2. The inter-examiner ICC value was 0.91 (PPD) and 0.97 (CAL). The values were assessed on six sites of all present teeth (except to 3rd molars) using a periodontal probe (UNC 15, Trinity, Trinity-Odontologia, São Paulo, SP, Brazil). The periodontitis diagnosis was based on the full-mouth estimated PPD and CAL values, according to the Center for Disease Control and Prevention in association with the American Academy of Periodontology (CDC/AAP) criteria ^1^.

### Sample collection

The subgingival biofilm samples were collected from the six deeper pockets of non-adjacent teeth. Each tooth was isolated with cotton rolls, and sterile gauze was used to remove the supragingival bioﬁlm. A sterile n^o^ 35 endodontic paper point (AllPrime, Meta Biomed Co. Ltd., Heungdeok-gu, South Korea) was inserted into the most apical part of each pocket and was left *in situ* for 30s. The points were then immediately transferred to two sterile, dry microtubes (Eppendorf do Brasil, São Paulo, SP, Brazil). Points were transferred for storage at -70 ⁰C until processing. The time between sample collection and storage in a freezer ranged from 40 to 60 min.

### Microbiological analysis

The DNA were extracted using a commercial kit for detection of bacterial colonization (Ludwig Biotecnologia, Porto Alegre, RS, Brazil) following the manufacturer’s protocol. All samples were analyzed for DNA quantity and purity based on optical density in a spectrophotometer (Pharmacia Biotech, DE, USA). Sufficient samples had an absorbance reading of 260 nm and an initial concentration between 0.5 and 0.6 µg/µl^-1^ for the relationship between absorbances at 260/280 nm.

Microbiological analysis of the periodontal pathogens with DNA of the standard strains of *A. actinomycetemcomitans* (ATCC 33384), *P. intermedia* (ATCC 25611), *F. nucleatum* (ATCC 49256), and *P. gingivalis* (ATCC 33277) were carried out using real time polymerase chain reaction analysis (RT-PCR) in the Biochemistry Research and Molecular Biology of Microorganisms Laboratory, UFPel. The primer sequences were the following: *P. gingivalis* forward TGCAACT TGCCTTACAGAGGG and reverse ACTCGTATCGCCCGTTATTC; *A. actinomycetemcomitans* forward CAAGTGTGATTAGGTAGTTGGTGGG and reverse CCTTC CTCATCACCGAAAGAA; *P. intermedia* forward CCACATATGGCATCTGAG GTG and reverse TCAATCTGCACGCTACTTGG; and *F. nucleatum* forward GAAGAAACAAATGACGGTAACAAC and reverse GTCATCCCCAC CTTCCTCCT (IDT", Integrated DNA Technologies, Iowa, USA).

The oral samples’ DNAs were amplified and marked with the Syber Green Mix/ROX reagent (Sigma-Aldrich, Foster City, California, USA) in RT-PCR reactions conducted using the Step One^TM^ Real-Time PCR System (Image Quant 100 - GE Healthcare®, Piscataway, New Jersey, USA) following the protocol used by Casarin et al. ^14^. All reactions were performed in a total volume of 15µL containing 7.65µL Syber Green mix/ROX (Sigma-Aldrich, Foster City, California, USA), 0.30μl of each pair IDT^®^ oligonucleotide primers (Integrated DNA technologies, Iowa, USA) and 6.75μl DNA extracted from the sample. All reactions were carried out in the presence of positive control, containing genomic DNA specific to the bacteria under analysis, and negative control, without DNA. Thermocycling was conducted as follows: 94°C for 5 min, followed by 40 cycles of denaturation at 94°C for 45 s, annealing at 55.4°C (*P. gingivalis*), 55°C (*A. actinomycetemcomitans*) and 58°C (*F. nucleatum* and *P. intermedia*) for 1 min, extension at 72°C for 1 min and a final extension at 72°C for 10 min. After the RT-PCR, it was performed a dissociation curve (melting curve) with a temperature between 60ºC and 95ºC to determine the specificity of the RT-PCR. All reactions were accomplished in MicroAmp™ Fast Optical 48-Well Reaction Plate tubes (Applied Biosystems®, Concord, Ontario, Canada). After optimization, four dilutions were selected for the standard curve with DNA extracted from the pure bacterial culture. This standard curve of known concentration was used to convert the cycle threshold score obtained from the samples into exact numbers of DNA concentrations. The data were analyzed using the Step One^TM^ program (Applied Biosystems®, Concord, Ontario, Canada).

### Statistical analysis

The statistical power for the analysis was calculated. It was considered an alpha error probability of 0.05, a mean score of log10 of *P. gingivalis* counts (standard deviation [SD]) of the Normotensive group of 9.51 (SD 0.34) and mean of the hypertensive group of 9.77 (SD 0.25), resulting in a sample power of 70.4%.

The primary outcome of the study was the comparison of total count for each bacterial species analyzed between hypertensive and normotensive patients. The independent variables were related to sociodemographic factors and systemic conditions. Sociodemographic factors were evaluated as follows: age in years (median ≤ 55 / > 55), gender (female/males), self-reported ethnicity (white/non-white), education level (>8/≤8 completed years of study - corresponding to a primary school education in Brazil), household income based on the Brazilian monthly minimum wage (BMMW - R$954.00; equivalent to approximately US$250 during the study period): >1 BMMW / ≤1 BMMW, and cigarette smoking status (never smoker/former smoker). The independent variables related to the systemic conditions were investigated by using the following cut-off points as increased levels: fasting blood glucose >99 mg/dL; triglycerides ≥150 mg/dL; HDL cholesterol ≥50 mg/dL, LDL cholesterol >130 mg/dL and total cholesterol ≥200 mg/dL; CRP ≥6 mg/L; fibrinogen ≥300 mg/dL; body mass index (BMI) ≥30.

The data were expressed as mean, standard deviation, and median values. The Shapiro-Wilk test was used to determine the distribution (normal or non-normal) of the data. As non-normal distribution was determined, the characteristics of the sample were compared using the Mann-Whitney and Fisher exact test for clinical and microbiological comparison. Statistical analyses were performed with the software STATA 14 (Stata Corporation; College Station, TX, USA) and the level of significance was 5%.

## Results

### Patients

**Figure 1:**
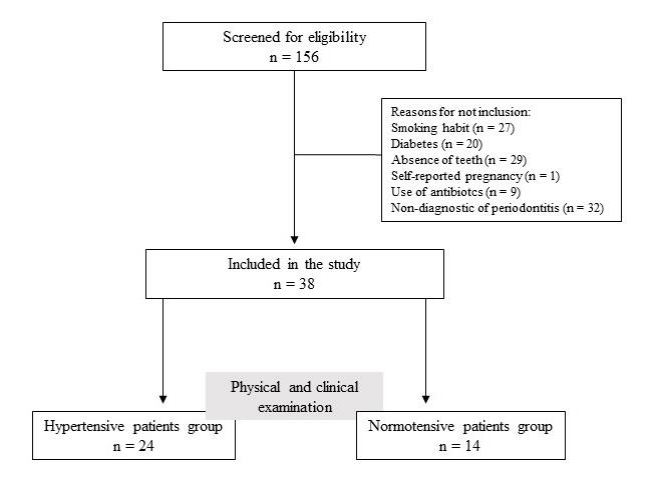
Flowchart of the study.

From 156 individuals evaluated for eligibility, 38 patients were included until this moment. The reasons for not inclusion were smoking habit (n = 27), diabetes (n = 20), absence of teeth (n = 29), self-reported pregnancy (n = 1), use of antibiotics in the last 3 months (n = 9), and non-diagnostic of periodontitis (n = 32). Among the 38 patients, 24 were hypertensive and 14 were normotensive patients. Figure 1 describes the flowchart of the study.

Table 1 presents the demographic, socioeconomic, and behavioral characteristics of hypertensive and normotensive patients. The normotensive group was mostly ≤55 years old (n=11), while the hypertensive group was mainly >55 (n=14). Both groups were composed, mostly, of white (NG: 13; HG: 18), female (NG: 10; HG: 15), and never smokers (NG: 9; HG: 15) individuals. Although some patients did not answer about income, most of the responses were in the range of around one BMMW.

Systemic conditions and CRP levels were assessed as independent variables according to establish cut-off points as increased levels, and a similar distribution could be observed in both groups. The use of medication was mainly reported by the HG for antihypertensive drugs (p=0.001), antidepressant drugs (p=0.041), and diuretic drugs (p=0.003). Also, the blood pressure was slightly higher in the hypertensive group (128.2mmHg ± 12.78 / 80.7mmHg ± 8.63) compared to the normotensive group (118.5mmHg ± 12.17 / 70.21mmHg ± 9.29) (Table 2).

### Periodontal condition

Comparing the periodontal parameters of the two groups, hypertensive individuals demonstrated higher percentage of VPI (p=0.048), MBI (p=0.019), PRF (p=0.023), and sites with 1-3 mm CAL (p=0.031). Besides, statistical difference was observed between the groups regarding the mean CAL value at all evaluated sites (p=0.022). Concerning PPD, BOP and periodontitis diagnosis, there were no statistical differences between the groups (Table 3). The PPD averages of the biofilm collection sites were compared and did not show statistical differences between groups for any of the sites (p>0.05). The average values at the six sites were as follows: Mean PPD 1 (NG: 3.71 ± 1.48; HG: 3.58 ± 1.01; p = 0.946), Mean PPD 2 (NG: 3.42 ± 1.15; HG: 3.66 ± 1.3; p = 0.478), Mean PPD 3 (NG: 3.35 ± 0.92; HG: 3.7 ± 1.26; p = 0.520), Mean PPD 4 (NG: 3.28 ± 0.91; HG: 3.54 ± 0.93; p = 0.351), Mean PPD 5 (NG: 3.35 ± 1.27; HG: 3.61 ± 0.97; p = 0.292) and Mean PPD 6 (NG: 3.35 ± 1.33; HG: 3.52 ± 0.98; p = 0.401).

**Table 1:** Demographic, socio-economic and behavioral characteristics of normotensive and hypertensive patients (n=38).

	Normotensive (n=14)	Hypertensive (n=24)
	n (%)	n (%)
Age (years)		
≤55	11 (78.6)	10 (41.7)
>55	3 (21.4)	14 (58.3)
Sex		
Female	10 (71.4)	15 (62.5)
Male	4 (28.6)	9 (37.5)
Smoking abstinence*		
Never smoker	9 (64.3)	15 (62.5)
Former smoker	5 (35.7)	6 (25)
Ethnicity*		
White	13 (92.9)	18 (75)
Black	1 (7.1)	4 (16.7)
Schooling (years)		
>8	12 (85.7)	12 (50)
≤8	2 (14.3)	12 (50)
Income* (BMMW)^a^		
>1	12 (85.7)	12 (50)
≤1	2 (14.3)	7 (29.2)

*There was missing data regarding smoking abstinence (three hypertensive patients), ethnicity (two hypertensive patients) and income (five hypertensive patients); ^a^BMMW: Brazilian monthly minimum wage ≈ R$ 954 during the study period.

**Table 2:** Systemic conditions and medication use by normotensive and hypertensive patients (n=38).

	Normotensive (n=14) n (%)	Hypertensive (n=24) n (%)	P value
Depression^#^	3 (21.4)	4 (16.7)	0.761
Obesity (BMI^a^ >30) ^#^	1 (7.1)	3 (12.5)	0.604
Medication^#^			
Antihypertensive^b^	0	23 (95.8)	0.001
Antidepressant^c^	0	6 (25)	0.041
Diuretics^d^	0	11 (45.8)	0.003
Anticoagulant^e^	0	2 (8.3)	0.267
Cholesterol^f^	1 (7.1)	5 (20.8)	0.264
Blood exam, *mean ± sd* ^*^			
Triglycerides	109.3 ± 60.8	114 ± 65.3	0.739
Fasting blood glucose	84.9 ± 7.1	90.2 ± 8.3	0.051
Fibrinogen	265.1 ± 64.9	279.1 ± 61.0	0.449
C-reactive protein	0.2 ± 0.6	0.1 ± 0.3	0.532
HDL Cholesterol	53.6 ± 10.1	57.2 ± 13.2	0.332
LDL Cholesterol	125.4 ± 15.3	119.2 ± 34.8	0.355
Total Cholesterol	200.6 ± 15.2	197.6 ± 41.6	0.606
Blood pressure (mmHg), *mean ± sd* ^*^			
Systolic	118.5 ± 12.17	128.2 ± 12.78	0.024
Diastolic	70.21 ± 9.29	80.7 ± 8.63	0.001

^a^ BMI: Body mass index; ^b^Losartan, Atenolol, Amlodipine, Enalapril, Captopril, Carvedilol, Digoxin, Propranolol, Lisinopril, Metoprolol succinate, Valsartan; ^c^Clonazepam, Paroxetine, Nortriptyline, Bupropion, Sertraline; ^d^Hydrochlorothiazide, Furosemide, Spironolactone; ^e^Warfarin, AAS; ^f^Simvastatin; Rosuvastatin. ^*^Mann-whitney test; ^#^Fisher exact test

**Table 3:** Comparisons of periodontal clinical variables among normotensive and hypertensive patients (n=38).

	Normotensive (median, 25^th^P - 75^th^P) (n = 14)	Hypertensive (median, 25^th^P - 75^th^P) (n = 24)	P value
VPI (%)^*^	24.29 (19.13-34.72)	41.66 (23.61-51.38)	0.048
MBI (%)^*^	4.16 (1.66-5.35)	8.33 (5.30-15.87)	0.019
PRF (%)^*^	19.93 (11.72-30-76)	31.88 (22.91-40.57)	0.023
BOP (%)^*^	7.04 (1.87-19.59)	12.58 (5.98-15.41)	0.184
PPD 1-3 mm (%)^*^	99.10 (90.33-99.90)	96.25 (92.89-99.70)	0.536
PPD 4-5 mm (%)^*^	4.32 (1.79-20.92)	4.85 (1.85-7.8)	0.505
PPD ≥6 mm (%)^*^	1.68 (0.95-6.88)	1.67 (0.69-1.75)	0.723
PPD (mm)^*^	1.89 (1.87-2.35)	1.95 (1.81-2.35)	0.963
CAL 1-3 mm (%)^*^	25.71 (14.88-30.76)	46.87 (26.27-61.94)	0.031
CAL 4-5 mm (%)^*^	5.33 (3.48-17.06)	15.27 (5.55-29.16)	0.154
CAL ≥6 mm (%)^*^	3.11 (0.69-3.33)	2.17 (2.08-4.62)	0.826
CAL (mm)^*^	0.95 (0.60-1.80)	2.07 (1.12-2.91)	0.022
Periodontitis diagnoses, *n (%)* ^#^			
Mild	2 (14.28)	2 (8.33)	
Moderate	10 (71.42)	15 (62.5)	0.589
Severe	2 (14.28)	7 (29.16)	

25^th^P - 75^th^P: 25^th^ Percentile and 75^th^ Percentile; VPI, visible plaque index; MBI; marginal bleeding index; PRF, plaque retention factors; BOP, bleeding on probing; PPD, probing pocket depth; CAL, clinical attachment loss. *Mann-whitney test; # Fisher exact test, ¶ Center for Disease Control and Prevention in association with the American Academy of Periodontology (CDC/AAP).

### Microbiological analysis

**Figure 2:**
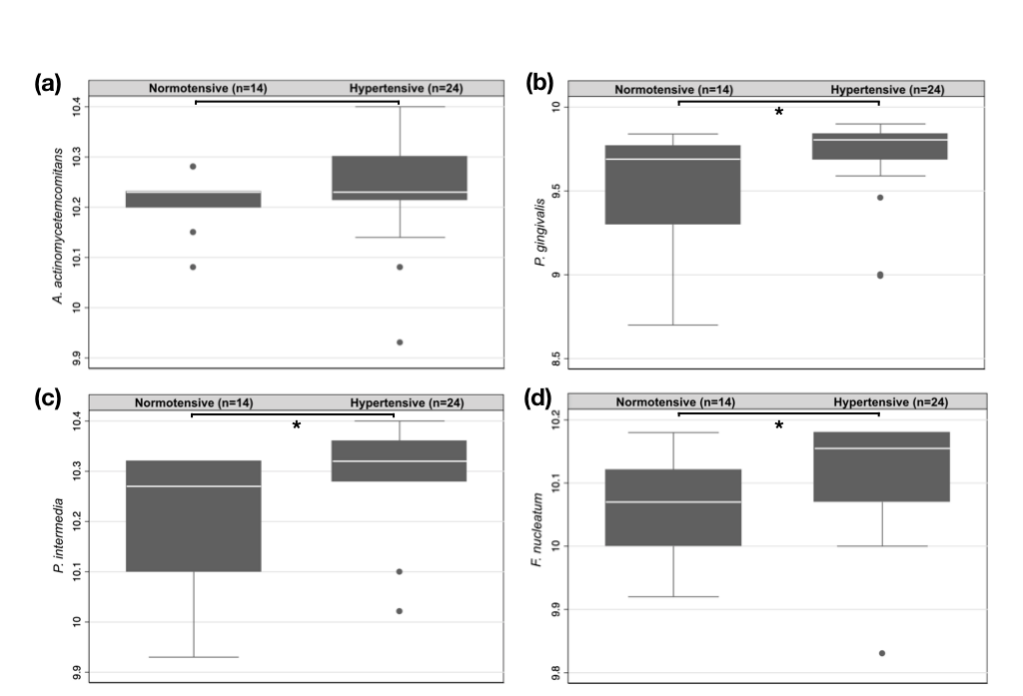
Bacteria distribution count (log_10_) in normotensive and hypertensive individuals. (a) *A. actinomycetemcomitans* (b) *P. gingivalis* (c) *P. intermedia* (d) *F. nucleatum* (CFU/µL). *Mann-Whitney test

Samples from all patients included in the study were used for microbiological analysis and were positive for all four bacteria species. The comparison of bacterial strains counts between normotensive and hypertensive groups showed significant differences for the strains: *P. gingivalis* (NG: 9.51 ± 0.34; HG: 9.77 ± 0.25; p = 0.015), *P. intermedia* (NG: 10.20 ± 0.13; HG: 10.31 ± 0.10; p = 0.003), and *F. nucleatum* (NG: 10.05 ± 0.09; HG: 10.12 ± 0.08; p = 0.022). The count of strain *A. actinomycetemcomitans* did not show any significant difference between groups (NG: 10.21 ± 0.05; HG: 10.23 ± 0.10; p = 0.246). Figure 2 exposes the comparison of each bacterial species in log_10_ in the NG and HG.

## Discussion

This pilot study aimed to evaluate and compare microbiological counts of hypertensive and normotensive patients with periodontitis, to understand possible changes in these outcomes associated with the presence of arterial hypertension. The results revealed a significant association between arterial hypertension and a greater quantity of the periodontopathogenic bacterial species of *P. gingivalis, P. intermedia,* and *F. nucleatum* (p<0.05) compared to a normotensive group. The periodontal condition was similar in both groups, with differences only in the VPI, MBI, PRF, mean CAL and CAL 1-3mm for the hypertensive group (p<0.05). No significant differences were observed in *A. actinomycetemcomitans* counts, PPD, BOP or in general health and systemic inflammatory markers between the groups.

The microbiological results showed data that corroborates the existing evidence. A recent literature review reported that among the oral pathogens related to systemic diseases, the species *A. actinomycetemcomitans, P. intermedia,* and *P. gingivalis* are those related to damage to the cardiovascular system ^5^. Furthermore, results of a cross-sectional study using data from 7,928 participants from the Third National Health and Nutrition Examination Survey - NHANES III ^6^, showed that participants with high blood pressure status (BP≥130/80 mmHg) had higher serum antibodies against all periodontal microorganisms investigated in the present study (*A. actinomycetemcomitans, P. intermedia, F. nucleatum* and *P. gingivalis*) when compared to individuals with BP<130/80 mmHg.

The role of *P. gingivalis* in the progression of periodontal diseases is extremely relevant, as this pathogen can spread through the upper layers of gingival epithelial cells and penetrate the basement membrane and connective tissue, contributing to the loss of periodontal attachment ^15^. The most expressive number of *P. gingivalis* observed in hypertensive patients agree with findings from previous studies, which demonstrate that *P. gingivalis* has the potential to evade innate immune detection via Toll-like receptor (TLR)-4, facilitating chronic vascular inflammation ^16^. A case-control study involving hypertensive and normotensive pregnant women evaluated placental biopsies using the PCR technique and found an association between *P. gingivalis* in placental tissues and hypertensive disorders (OR: 7.59, p = 0.019, CI 95% 1.39-41.51) ^17^. In this context, the higher *P. gingivalis* count observed in the hypertensive group could be associated with the hypertension condition.

It is approached that the red-complex bacteria (*Porphyromonas gingivalis, Tannerella forsythia,* and *Treponema denticola*), accompanied by the *Prevotella*, *Fusonucleatum* species and *A. actinomycetemcomitan* are mainly present in deeper periodontal pockets described in patients with periodontal disease and are involved in the onset and progression of the periodontitis ^18^. This study assessed four bacteria, two of orange complex (*P. intermedia and F. nucleatum*), one of red complex (*P. gingivalis*) and one (*A. actinomycetemcomitans*) strongly associated in the past with aggressive periodontitis ^19^ ^,^ ^20^ ^,^ ^21^. Considering that the subgingival biofilm samples were collected from the sites with the deepest periodontal pockets and that there was no significant difference in the PPD parameter between the groups, the differences found in bacterial load between hypertensive and normotensive individuals may be related to other factors. Although the limitations of the design of the present study do not allow the understanding of these factors, the results corroborate with previous studies that report that the composition of oral bacteria and blood pressure can be associated ^22^ ^,^ ^23^ ^,^ ^24^.

The present study did not demonstrate significant differences related to the count of *A. actinomycetemcomitans* between the groups, and in this respect, some points could be discussed. These pathogens were for many years more commonly related to periodontal infections affecting young individuals, being associated in the past, with the prevalence of juvenile / aggressive periodontitis ^25^. Besides the more pronounced expression of *A. actinomycetemcomitans* in adolescents and young adults, the literature reports that the proportion of the population harboring *A. actinomycetemcomitans* can vary in different geographical areas, in different clinical presentations of periodontitis and according to the patient's susceptibility ^25^. Another important factor to be considered is related to the colonization and behavior of these periodontal pathogens. The variable colonization pattern of this bacterial species could be related to the absence of differences in its counts between groups.

The use of different types of drugs reported in this study, mainly by hypertensive patients, should be considered, especially due to the effects that these drugs can have on the periodontium. Several drugs can cause specific changes in periodontal tissues, influencing the clinical presentation or progression of periodontal diseases ^26^. To understand the association of periodontal diseases with systemic diseases, it’s important to note that the frequency of medication intake, besides reflecting on the severity of the systemic disease, may indirectly represent the systemic inflammatory burden in chronic-inflammatory diseases ^27^. In the present study, all patients included in the HG used medication to control blood pressure. In this sense, the use of angiotensin-converting enzyme inhibitors (ACEi) (lisinopril, losartan, enalapril), β-blockers (atenolol, carvedilol), β-adrenoceptor antagonists (propranolol), and calcium channel blockers (amlodipine) was reported by hypertensive patients, which is in agreement with the current literature, that describes these drugs classes as the most used to control hypertension, together with diuretics ^26^ ^,^ ^27^.

Furthermore, emerging evidence suggests that ACE affects diverse biological processes, including several aspects of the immune response. Animal studies report that ACE overexpression could upregulate the immune responses of monocytes, macrophages, and neutrophils ^28^ and facilitate the host's defense against bacterial infections ^28^ ^,^ ^29^. In this context, it has been described that ACEi seems to be harmful to the neutrophilic function, and their use should be indicated with caution in the treatment of certain individuals ^28^. Despite the incipient nature of this evidence, the influence that ACEi could have on bacterial counts in hypertensive individuals may be considered in understanding the differences between groups.

Additionally, the effect of antihypertensives on periodontal tissues has also been reported in experimental studies ^30^. It’s described that low dosages of propranolol could suppress bone resorption by inhibiting RANKL-mediated osteoclastogenesis, as well inflammatory markers in experimental periodontitis in rats ^31^. In the same reasoning, rats treated with carvedilol with induced periodontitis had reduced levels of pro-inflammatory cytokines (IL-1 β TNF-α), as well as MMP-2, MMP-9, RANK, RANKL, COX-2, and OPG, demonstrating that the medication can influence bone formation / destruction and anti-inflammatory activity in periodontitis ^30^.

It is important to highlight that this pilot study presents data from an incomplete sample, which has a disparity in size between groups. This investigation is also limited by its cross-sectional design and the impossibility of blinding the examiner, which restricts the extrapolation of the results. However, the interdisciplinary character of this research contributes to the development of a broader view of the characteristics of periodontitis in systemically compromised individuals. Also, data from periodontal examinations performed in the full mouth, and the use of a highly sensitive technique for bacteria count should be seen as relevant points of the present study.

Within the limits of this study, it can be concluded that the presence of arterial hypertension may be associated with a greater quantity of periodontopathogenic bacterial of some species in individuals with periodontitis. Nevertheless, longitudinal studies investigating other bacteria species, from the large number involved in the development and evolution of periodontitis, should be conducted to determine the impact of hypertension on the oral microbiota and periodontal status. As well, clinical trials are needed to determine whether periodontal treatment could benefit the clinical and microbiological parameters in hypertensive patients.
